# Allogeneic Chimeric Antigen Receptor Therapy in Lymphoma

**DOI:** 10.1007/s11864-021-00920-6

**Published:** 2022-02-25

**Authors:** Arushi Khurana, Yi Lin

**Affiliations:** Division of Hematology, Mayo Clinic, 200 First Street, SW, Rochester, MN 55905 USA

**Keywords:** CAR-T, Allogeneic CAR-T, Non-Hodgkin lymphoma

## Abstract

The therapeutic armamentarium has significantly expanded since the approval of various CD19-targeting chimeric antigen receptor T cell (CAR-T) therapies in non-Hodgkin lymphoma (NHL). These CAR-Ts are patient-specific and require a complex, resource, and time-consuming process. While this appears promising, autologous CAR-Ts are limited due to the lack of accessibility, manufacturing delays, and variable product quality. To overcome these, allogeneic (allo) CARs from healthy donors appear appealing. These can be immediately available as “off the shelf” ready-to-use products of standardized and superior quality exempt from the effects of an immunosuppressive tumor microenvironment and prior treatments, and potentially with lower healthcare utilization using industrialized scale production. Allogeneic CARs, however, are not devoid of complications and require genomic editing, especially with αβ T cells to avoid graft versus host disease (GvHD) and allo-rejection by the recipient’s immune system. Tools for genomic editing such as TALEN and CRISPR provide promise to develop truly “off the shelf” universal CARs and further advance the field of cellular immunotherapy. Several allogeneic CARs are currently in early phase clinical trials, and preliminary data is encouraging. Longer follow-up is required to truly assess the feasibility and safety of these techniques in the patients. This review focuses on the strategies for developing allogeneic CARs along with cell sources and clinical experience thus far in lymphoma.

## Introduction

Chimeric antigen receptor T cell (CAR-T) therapy has been transformative for the treatment of relapsed/refractory (R/R) large B cell lymphoma (LBCL) patients who have relapsed post 2 lines of therapy or autologous stem cell transplant (ASCT). These patients were otherwise subject to dismal outcomes [1]. Since 2017, three different CAR-T products have been approved by the US Food and Drug Administration (FDA) for the treatment of R/R LBCL after two or more prior lines of therapy [2•, 3•, 4•]. All three boast a relatively similar durable response rate of around 40% at 2 years in the registration trials and real-world settings [5–8, 9]. Two additional FDA approvals for lymphoma in the last year have been for mantle cell lymphoma (brexucabtagene autoleucel) and follicular lymphoma (axicabtagene ciloleucel) [10, 11•]. All the commercially approved CAR-T therapies are directed against CD19, a protein that is frequently expressed in B cell lymphomas, and in a relatively higher proportion than some other targets such as CD22 or CD20 [12]. The commercial production of autologous CAR-T cells requires the collection of T cells from the patient (leukapheresis), followed by activation and transduction with the CAR constructs using viral vectors, and eventually expansion before reinfusion into the patient after a short course of lymphodepleting chemotherapy [12]. This bespoke manufacturing process can take up to 2–5 weeks, a delay that may be particularly problematic for those with aggressive and rapidly proliferative lymphomas. Up to 30% of patients could not receive tisagenlecleucel CAR-T therapy in the JULIET trial, most of whom died due to progressive disease as the median time from leukapheresis to infusion was 54 days [13]. Additionally, while waiting for the cells to be manufactured, some patients require additional bridging therapy to maintain disease control which is associated with poor outcomes [6•, 9, 14]. Delay in CAR-T therapy and increase in the lines of therapy before CAR-T have both been shown to worsen outcomes along with an increase in tumor burden, markers of inflammation, and doubling time of CAR-T cells [15, 16, 17]. Autologous CAR-Ts are expensive and laborious to manufacture due to patient specificity, vary in quality based on patient’s disease factors and prior treatments, limit redosing due to small-scale individual production, and may not be effective if generated from dysfunctional T cells due to an immunosuppressive tumor microenvironment seen in many lymphoma types [15, 18, 19]. To overcome these issues related to cost, accessibility, efficacy, and efficiency, “off the shelf” allogeneic (allo) CAR-Ts are being explored. While they resolve some of the challenges encountered with autologous CAR-T, they also come with inherent hurdles such as the risk of graft versus host disease (GvHD) and rejection of the CAR-Ts by the host terminating their treatment effect [20, 21]. We herein review the different strategies of generating, overcoming challenges associated with allo CAR-Ts, their clinical experience in lymphoma, and implications of their use.

## Strategies to generate “off the shelf” or universal allogeneic CAR-T

**Fig. 1:**
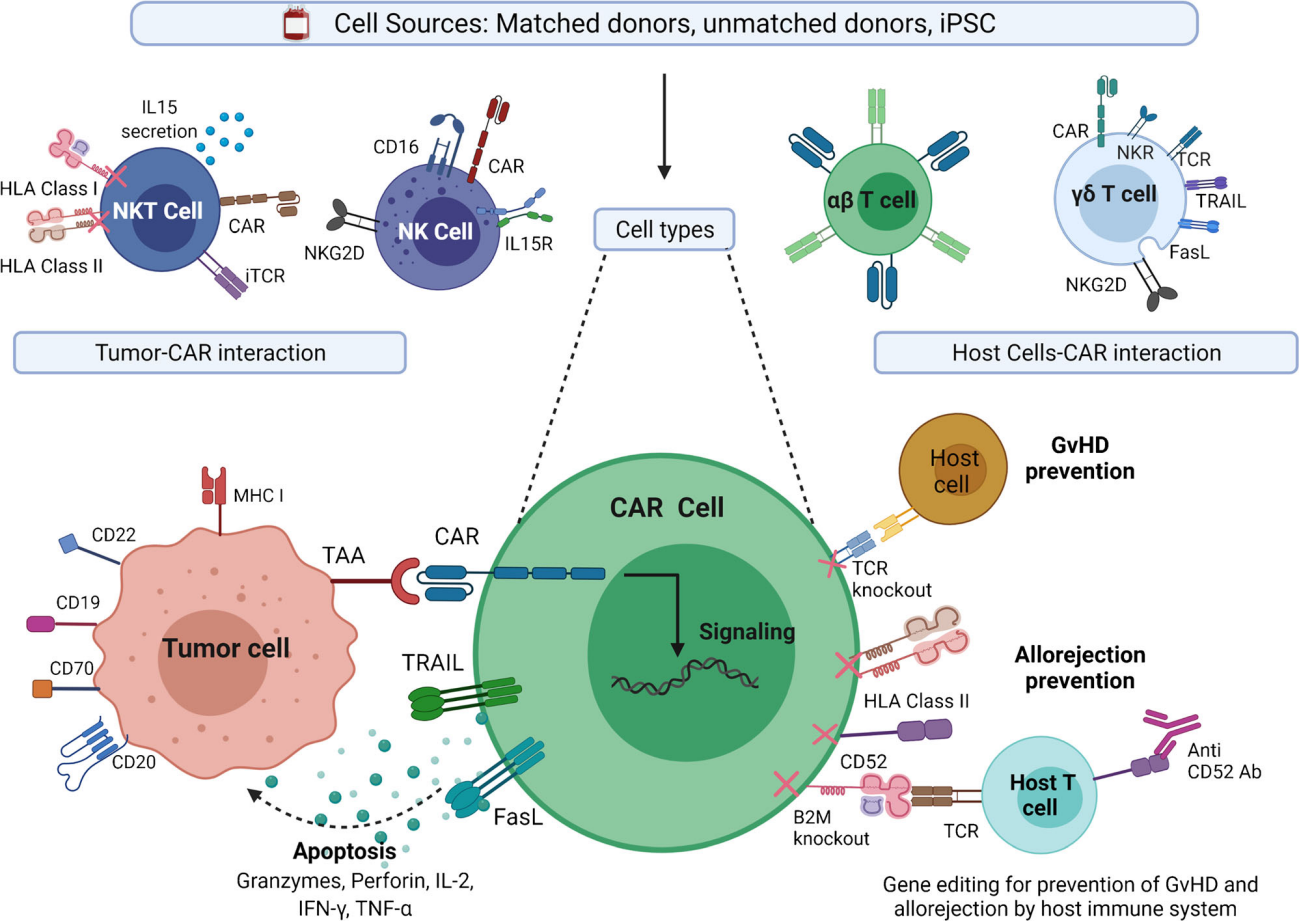
Various allogeneic CARs with genomic editing techniques (abbreviations: CAR, chimeric antigen receptor; TAA, tumor associated antigen; B2M, β2 microglobulin; Ab, antibody; TCR, T cell receptor; FasL, Fas ligand; iPSC, induced pluripotent stem cells; TRAIL, tumor necrosis factor-related apoptosis-inducing ligand; IL, interleukin; NKG2D, natural killer group 2 member D).

For a successful generation of “off the shelf” or “universal” allo CAR-Ts, two important hurdles need solutions. First is the GvHD, which is mediated by the alloreactivity of the T cell receptor (TCR) on the CAR-T cells, which recognize the recipient’s human leukocyte antigens (HLA) as foreign and mount an immune attack. Contrary to the first issue is the risk of rejection of allo CAR-Ts by the recipient’s immune system, resulting in their destruction. An essential requirement for the allo CAR-Ts to succeed is their persistence in the recipient, and lack thereof would not allow for sustained responses. While this does not apply to autologous CAR-Ts as seen in aggressive NHL with CD28 co-stimulatory domain in axi-cel, allo CAR-Ts are subject to rapid elimination by the host immune system, further shortening their persistence. Additionally, with allo CAR-Ts, there is also a risk for alloimmunization, which may lead to issues with redosing similar products due to donor-specific antibodies (DSA). Similar problems due to DSA have been encountered in patients undergoing solid organ transplantation and haploidentical stem cell transplantation [22, 23]. Comparison of features and implications between autologous and allogeneic CAR-T is outlined in Table 1. Several strategies to overcome these issues outlined above are in development and discussed here (Fig. 1).

**Table 1:** Differences between the autologous and allogenic CAR-Ts

Characteristic	Autologous	Allogeneic
Cell source	Self	Non-self healthy donor, iPSC derived
Production time	2–5 weeks	Ready to use/off the shelf
Cell type	Autologous T cells (may or may not be manipulated for T cell composition)	Gene-edited αβ T cells, γδ T cells, NK cells, iNKT cells
Manufacturing process	• Individualized manufacturing for each patient • Reduced accessibility due to manufacturing wait and potential impact of patients’ native cells on manufacturing success • High variability in product composition due to inherent patient heterogeneity in T cell composition and immune profile	• Single manufacturing from one donor or cell source potentially used to treat many patients • Pre-made and ready to use to remove the manufacturing wait time • Possible standardization and control over T cell composition in the product
Side Effects	• Cytokine release syndrome • Immune effector cell-associated neurotoxicity syndrome • B cell aplasia • CAR gene editing-related oncogenic potential	• Same as autologous PLUS • More immune suppression and risk for infection from more intense lymphodepletion for prevention of allo rejection • Graft versus host disease • Allo rejection of the infusion product
Redosing	Response with the same CAR-T unfavorable in aggressive lymphoma, possibly more promising in FL More studies needed to understand mechanisms of relapse and resistance to redosing	Benefit to be investigated Risk of alloimmunization—donor-specific antibodies
Persistence	Months to years	Weeks to months
Cost	High	Unknown (projected to be lower)

### Developing CAR-T from a stem cell transplant donor

αβ T cells are the main drivers of GvHD in allogeneic stem cell transplant recipients [24–26, 27–29]. One strategy to decrease the risk of GvHD for patients who have already received an allogeneic stem cell transplantation and have subsequently relapsed is to derive CAR-Ts from the same donor T cells. This was attempted in a clinical trial where 20 patients with B cell malignancies that had relapsed after allo stem cell transplant received a single infusion of donor-derived CAR-Ts targeting CD19 with no prior lymphodepletion chemotherapy. Eight of the 20 patients achieved a response with 6 complete remissions (CR). No GvHD was reported, and CAR-T expansion was higher for those with a response than non-responders, and no CAR-T cells persisted beyond 3 weeks [30–32]. This study proved the feasibility, safety, and initial efficacy of such an approach albeit, with small patient numbers. Additionally, it highlights the use of allo CAR-Ts to generate a graft versus leukemia/lymphoma effect without significantly increasing the risk for GvHD. Another recent phase I clinical trial of 10 patients with relapsed or refractory B-acute lymphoblastic leukemia or aggressive lymphoma described the generation of CD19-targeting CAR-T from HLA-matched sibling donors using the high-capacity piggy-Bac transposon method for genetic modification. At a median follow-up of 18 months, 5 patients were in CR [33]. Interestingly, 2 of the 10 patients developed malignant lymphoma derived from CAR gene-modified T cells, and therefore, this strategy requires further follow-up [34]. It remains patient-specific and limited to those who have previously undergone allo stem cell transplant with available T cells and not with a history of GvHD.

### Selecting non-alloreactive T cells as a source for CAR-T

#### Virus-specific T cells

These have been used for several years now in patients with allo stem cell transplants for the treatment of viral infections without additional risk of GvHD [35–37]. This is thought to be due to the restricted repertoire of these memory T cells. Another disease where this approach has been promising is the Epstein-Barr virus (EBV)-associated malignancies, such as post-transplant associated lymphoproliferative disorder (PTLD). This approach combines the antigen specificity of CAR-Ts with the TCR specificity towards EBV. Recently, Prockop and colleagues reported on a single-center cohort of 46 patients with rituximab refractory PTLD developing after allo transplant (*n* = 33) or solid organ transplant (*n* = 13) who were treated with banked third-party partially HLA-matched EBV-specific cytotoxic T cells. Each treatment cycle consisted of 3 weekly infusions, followed by a 3-week observation period. This product named tabelecleucel showed a 1-year estimated overall survival (OS) for patients with PTLD following allo stem cell transplant and solid organ transplant of 68 and 64%, respectively. The median OS for post allo transplant PTLD cohort had not been reached after a median follow-up of 23.3 months. The median OS for post solid organ transplant PTLD cohort was 21.3 months. Treatment-related serious adverse events were reported in 1 patient each in both the allo and solid organ transplant cohorts, respectively [38, 39, 40]. Tabelecleucel is currently in phase 2 (NCT04554914) and 3 trials for EBV+ PTLD following allo stem cell transplant post rituximab failure (NCT03392142, ALLELE study) and solid organ transplant after the failure of rituximab or rituximab and chemotherapy (NCT03394365).

#### Memory T cells

These have a more restricted TCR repertoire as compared to naïve T cells and are therefore associated with less GvHD [41, 42]. They have shown to generate an effective anti-tumor response both in the preclinical and in autologous setting in non-Hodgkin lymphoma [43, 44]. However, the studies suggest a correlation between the presence of less-differentiated T cells, such as naïve T cells (T_n_), central memory T cells (T_cm_), and stem cell-like memory T cells (T_scm)_ with CAR-T efficacy. These less-differentiated T cell subsets are essential for in vivo expansion, survival, and persistence [45, 46, 47]. Various T cell subtypes exist with varied phenotypes and functions in the tumor microenvironment of lymphoma [18, 48, 49]. A T cell subset expressing CD8, CD45RA, and chemokine receptor 7 (CCR7) with features of T_n_ cells or T_scm_ cells was found to be associated with increased in vivo expansion of CAR-T cells in lymphoma [50]. Fraietta et al. performed genomic, phenotypic, and functional testing to identify the determinants of response in CD19-targeting autologous CAR-T in chronic lymphocytic leukemia (CLL) and BCMA targeting autologous CAR-T in multiple myeloma. Sustained remission was associated with lymphocytes that possessed memory-like characteristics and had CD27^+^CD45RO^-^CD8^+^ expressing T cells before CAR-T generation [51, 52]. Therefore, further research is required to better understand the ideal composition of various T cell subsets in the allo CAR-T product. Only selecting for T_em_ cells might lead to ineffective therapy. No trials of allogeneic CAR-T with this strategy are currently ongoing.

### Using genome editing of αβ T cells

Novel genome editing tools have paved the way to using conventional αβ T cells to generate allo CAR-T [20, 53, 54]. αβ TCR removal by disruption of the gene encoding for alpha chain (TRAC) is sufficient and essential for preventing TCR-dependent GvHD. Torikai et al. were the first to report the feasibility and efficacy of αβ TCR(neg) CD19CAR(pos) T cells using the zinc finger nucleases (ZFNs) for permanent deletion of α or β TCR chains in B cell malignancies [55•]. Another genome editing tool used by Poirot et al. transcription activator-like effector nuclease (TALEN) was able to perform multiplex gene editing to manufacture T cells deficient in expression of both αβ TCR and CD52 [56]. These T cells were unable to cause GvHD in a mouse model and were resistant to removal by alemtuzumab (anti-CD52 monoclonal antibody), which can be used to eliminate host T cells and hence avoid rejection. Cluster regularly interspaced short palindromic repeats (CRISPR)/Cas9 with its rapid and efficient multiplex genome editing have been recently used to generate allogeneic universal CAR-T with up to 4 disrupted genes [57, 58]. While these approaches are very promising, they are not free from consequences. They may not result in complete knock out of αβ TCR in all T cells resulting in GvHD [59]. Two, there is a risk of “off-target” genome cleavage resulting in unwanted gene translocations or inactivation that could potentially confer oncogenic potential on the CAR-Ts [56]. Three, these multiple genomic changes could result in increased toxicity, poor transduction efficacy, or depending on the gene disruption, a proliferation and survival advantage to the CAR-Ts [60].

Recently, CRISPR/Cas9 technology was used to integrate CAR construct into the TRAC locus, to ensure that TCR is inactivated at the same time as CAR introduction [61•, 62]. Other advantages of this approach are that it is safer without insertional mutagenesis-related adverse effects. The CAR expression is regulated by endogenous TCR promoter, preventing constant T cell activation, differentiation, and exhaustion. These advantages render more potent anti-tumor activity compared to the conventionally transduced CAR-Ts [61•, 63, 64]. UCART19 an allo CD19 CAR-T is currently under evaluation in adult and pediatric ALL with promising result and up to 50% of patients able to proceed to allogeneic transplant [65].

There is still the risk of allo-rejection with TCR-negative allogeneic CAR-Ts due to the recognition of non-self HLA by the recipient’s T cells if there is a mismatch. While strategies such as lymphodepletion and irradiation can help delay the rejection, the CAR-T persistence which is necessary for efficacy will be relatively short. For this, the removal of class I HLA molecules is required from the T cells, which can be achieved by disrupting the beta-2 microglobulin (β2M) gene locus [57, 66–68]. CD19 and CD70 (COBALT-LYM)-targeting CAR-Ts with CRISPR/Cas9 disrupted TRAC and β2M genes (double knockout) are currently under evaluation in B cell lymphomas and leukemias (NCT03166878, NCT04502446). Further triple knockout of HLA classes I and II and TRAC by CRISPR-based techniques has shown even better persistence of CAR-Ts in vivo when compared to double knockout [67]. While the HLA class I knockout prevents allo-rejection from T cells, it does not contain the allo CAR-Ts from potential rejection by NK cells. Avoiding recognition by NK cells can be achieved by expression of non-classical HLA molecules such as HLA-E and G [66, 69]. The use of HLA homozygous donors to generate a bank of universal allo CARs can also help prevent rejection due to HLA mismatch [70]. Genome editing techniques indeed open up a wide range of opportunities in the development of allo CAR-Ts; clinical studies are needed to determine their efficacy and safety for use.

### Using non-αβ TCR T cells for CAR approach

Several other immune cell types can generate CAR-based therapy if they possess cytotoxic properties and are easily accessible from sources such as peripheral blood mononuclear cells (PMBCs), or renewable stem cells.

Natural killer (NK) cells are an attractive alternative as they are highly cytolytic to cancer cells via granzyme B, perforin, and Fas ligand. They are an integral part of tumor immunosurveillance and are often found to be dysfunctional due to cancer’s immunosuppressive mechanisms for immune escape. Due to their innate anti-tumor activity, without causing GvHD, CAR-expressing NK is appealing. This has been shown to be feasible and active in in vitro and xenogeneic tumor models, as well as against patient-derived glioblastoma cell lines [71, 72]. However, NK cells exist in low numbers in peripheral blood and are less pliable to genetic manipulation which makes its use difficult [73, 74]. A strategy uses the NK92 cell line, an activated human NK cell line derived from an NHL patient [75, 76]. CAR-NK cells derived from the NK92 cell line targeting CD20 have shown efficacy against lymphomas in vitro [77]. NK-92 cell line has disadvantages as it is derived from a patient with NK lymphoma and has the potential for tumor engraftment post infusion. In addition, these cells are EBV positive, which carry various cytogenetic abnormalities. Due to these reasons, the NK92 cell line needs to be irradiated before infusion into patients, which can negatively impact its expansion and persistence, ultimately leading to low efficacy [78]. Another strategy using umbilical cord blood (UCB) to obtain NK cells has been used to create CD19 directed CAR-NK cells. These UCB-derived NK cells were transduced with a retroviral vector incorporating the genes for CAR-CD19, interleukin-15 (IL-15), and inducible caspase-9-based suicide gene (iCasp9). Liu et al. reported efficient killing of CD19-expressing cell lines, with marked prolongation of survival in a xenograft lymphoma murine model. IL-15 production by the transduced UCB-NK cells critically improved their function over controls. The iCasp9 suicide gene upon pharmacologic activation resulted in rapid elimination of the iCasp9/CAR.19/IL-15 UCB-NK cells, thereby incorporating a safety switch [79]. This approach was evaluated in a phase I/II clinical trial where HLA-mismatched UCB-derived CAR-NK cells targeting CD19 were infused in 11 patients with relapsed or refractory CD19-positive B cell NHL or CLL. There were no cytokine release syndrome (CRS), neurotoxicity, or GvHD, and no increase in inflammatory markers such as IL-6. Of the 11 patients, 8 (73%) had a response, with seven patients achieving a CR; however, the full clinical activities of the NK cells were difficult to interpret as many patients received subsequent systemic therapy or stem cell transplant as consolidation/maintenance. The CAR-NK cells were detectable in some subjects at low levels for up to 12 months post infusion [80•]. To overcome the limited amount of NK cell present even in the UCB, a Good Manufacturing Practice (GMP)-compliant procedure has been developed which reliably generates clinically relevant doses of GMP-grade NK cells from a UCB unit [81]. These studies support the use of NK cells as a source of allo CAR-Ts, and several products are currently in early phase clinical trials (Table 2).

**Table 2:** Ongoing clinical trials of allogeneic CARs in non-Hodgkin’s lymphoma

Trial ID/Phase	Company/product name	CAR Cell type	CAR Target	Gene Editing technology and modification	Location
**CAR-Ts with no TCR modification**					
NCT04176913/Phase I	Nanjing Legend Biotechnology Co., Ltd./LUCAR-20S	T-cells	CD20	Not specified	China
NCT04384393/Phase I	Fundamenta Therapeutics, Ltd./ThisCART19	T-cells	CD19	Not specified	China
NCT04601181/Phase I	Fundamenta Therapeutics, Ltd./ThisCART22	T-cells	CD22	Not specified	China
NCT04288726/Phase I	CD30.CAR-EBV Specific T cells	EBV-CTLs	CD30	Not specified	US
NCT01430390/Phase I	Memorial Sloan Kettering Cancer Center/ EBV-CTLs	EBV-CTLs	CD19	Not specified	US
NCT03768310/Phase I	Baylor College of Medicine	Multivirus-Specific Cytotoxic T Lymphocytes	CD19	Not specified	US
NCT02050347/Phase I (CARPASCIO study)	Baylor College of Medicine	Donor derived T-cells post Allo transplant	CD19	Not specified	US
NCT04538599/open label safety	He Huang, Zhejiang University/ RD13-01	T-cells	CD7	Not specified	China
**Genome edited CAR-Ts**					
NCT04264039/Phase I	Xinqiao Hospital of Chongqing; Gracell Biotechnology Shanghai Co., Ltd./Universal CD19CAR-T	T-cells	CD19	Not specified	China
NCT03398967/Phase I-II	Chinese PLA General Hospital/ Universal CRISPR-Cas9 Gene-Edited Dual CAR-T Cells	T-cells	CD19 and CD20/ CD22	CRISPR-Cas9 Gene-Editing, disruption of endogenous TCR and β2M genes	China
NCT04026100 /Phase I	Nanjing Bioheng Biotech Co., Ltd./CTA101	T-cells	CD19/CD22	CRISPR-Cas9 Gene-Editing TRAC region and CD52 gene disruption	China
NCT04035434/Phase I	CRISPR Therapeutics AG/CTX 110	T-cells	CD19	CRISPR-Cas9 Gene-Editing MHC-1 Knockout by β2M disruption and CAR inserted into TRAC locus	US, Australia, Germany, Canada
NCT04502446/Phase I (COBALT-LYM)	CRISPR Therapeutics AG/CTX130	T-cells	CD70	CRISPR-Cas9 Gene-Editing – CAR inserted into TRAC 9locus, MHC-1 Knockout by β2M disruption	US, Australia, Canada
NCT03939026/Phase I	Allogene Therapeutics/ALLO-501	T-cells	CD19	TRAC and CD52 Genes disruption by TALEN	US
NCT04416984/Phase I-II**	Allogene Therapeutics (ALPHA 2 study)/ALLO-501A	T-cells	CD19	TALEN mediated TCR and CD52 knockout	US
NCT03166878/Phase I-II	Chinese PLA General Hospital/UCART019	T-cells	CD19	CRISPR-Cas9 Gene-Editing disruption of endogenous TCR and β2M genes	China
NCT03229876/Safety and efficacy study	Bioray Laboratories/ CD19-UCART	T-cells	CD19	CRISPR-Cas9 Gene-Editing disruption of endogenous TCR and β2M genes	China
NCT03666000/Phase I-IIa (Cohort N)**	Precision BioSciences, Inc./ PBCAR0191	T-cells	CD19	ARCUS mediated gene editing – insertion of CAR at the TRAC gene locus	US
NCT04030195/Phase I-IIa	Precision BioSciences, Inc./ PBCAR20A	T-cells	CD20	CAR inserted into TCR locus	US
NCT04637763/Phase I (ANTLER study)	Caribou Biosciences, Inc./CB-010	T-cells	CD19	CRISPR-Cas9 Gene-Editing – CD19 CAR inserted into T cell genome, TRAC and PD 1 gene disrupted	US
NCT04264078/early phase I	Xinqiao Hospital of Chongqing, Gracell Biotechnology Shanghai Co., Ltd.	T-cells	CD7	CRISPR-Cas9 Gene-Editing – TRAC gene locus, and CD7 knockout to prevent fratricide	China
**Other “off the shelf” CARs**					
NCT03774654/Phase I (ANCHOR study)	Baylor College of Medicine/ CD19.CAR-aNKT cells KUR-502	NKT cells	CD19	Single gamma retroviral vector – CD19 CAR, IL15 and short hairpin RNA expression (downregulates HLA I, II)	US
NCT04245722/Phase I	Fate Therapeutics/FT596	iPSC derived NK cells	CD19		US
NCT04023071/Phase I-Ib	Fate Therapeutics/FT516	iPSC derived NK cells	non-cleavable CD16 (hnCD16) Fc receptor		US
NCT04673617/PhaseI-II	Artiva Biotherapeutics, Inc.	cord blood-derived NK cells	selected for B-KIR haplotype and the homozygous polymorphism of CD16		US
NCT04629729/Phase I	Fate Therapeutics/FT819	iPSC derived T cells	CD19	CAR inserted into TRAC locus	US
NCT02892695/Phase I-II	PersonGen BioTherapeutics (Suzhou) Co., Ltd./PCAR-119	NK cells	CD19	Derived from NK-92 cells line	China
NCT04887012/Phase I	Second Affiliated Hospital, School of Medicine, Zhejiang University/CAR NK019	HLA Haploidentical NK cells	CD19		China
NCT04639739/Early phase I	Chongqing Precision Biotech Co., Ltd	NK cells	CD19		China
NCT02656147/Phase I	Beijing Doing Biomedical Co., Ltd.	γδ T cells	CD19		China

Induced pluripotent stem cells (iPSCs) are another source for CARs. These can be genetically modified at a clonal level and used to create a homogeneous population of uniformly engineered T or NK cells. These have certain advantages, such as their unlimited division potential, and are more amenable to genetic modification [82]. Any somatic cell can be reprogrammed into iPSC; single clones from these cells can be expanded to create an iPSC cell line. T cells generated from iPSC still need genetic editing to prevent GvHD; however, once modified, a single cell can then be used to generate a clonal population of CAR-Ts with desired knockouts. Clinical trials are already underway using iPSC-derived CAR-Ts and CAR-NK cells in B cell lymphomas (NCT04023071, NCT04245722, NCT04629729; Table 2) [83].

NK T (NKT) cells are a subset of T cells that express NK cell markers. A subset of NKT cells called the “invariant NKT” (iNKT) cells expressed a highly restricted TCR, which can recognize lipid antigens presented by CD1d (HLA class I-like molecule) on B cells, antigen-presenting cells, and some epithelial cells [84, 85]. Allogeneic or donor iNKT cells have also shown to be protective against GvHD due to the production of IL-4 and promotion of a Th2-based immune response in preclinical models and clinical acute GvHD [86–88]. CD19-targeting CAR-iNKT cells showed a significantly improved efficacy over conventional CD19 CAR-Ts in a murine model of CD1d+ CD19+ B cell malignancy [89]. Protective effect against GvHD and improved efficacy over conventional CD19 CAR-Ts make this an explorable approach which is currently in phase I trial in B cell malignancies (NCT03774654).

γδ T cells are another potential candidate under investigation as allogeneic CAR-Ts. They represent a small percentage of circulating lymphocytes (1–5%), however, in abundance in certain tissue sites such as gut mucosa, reproductive organs, tongue, and skin [90–92]. γδ T cells form a key component of the innate immune system and tumor immunosurveillance and can target tumor antigens not recognized by αβ T cells [93]. Additionally, their tissue residency gives them an advantage in efficacy over αβ CAR-Ts which have poor penetrance into non-inflamed tumors. They are also less likely to induce GvHD as their TCR activation is not MHC restricted [94]. CD19-targeting CAR-T developed from γδ T cells has already shown efficacy in CD19+ B cell malignancies in vitro and in vivo and also in an early phase clinical trial (NCT02656147) [95].

## Clinical experience with allogeneic CAR-T in lymphoma

A list of completed and ongoing clinical trials using allo CAR-Ts in lymphoma is shown in Table 2. ALLO-501 is a genetically modified anti-CD19 CAR-T in which the *TCR α* gene is disrupted to reduce the risk of GvHD, and the *CD52* gene is disrupted to permit the use of ALLO-647 an anti-CD52 monoclonal antibody (mAb), for selective and prolonged host lymphodepletion. The phase I ALPHA study (NCT03939026) was first presented at the ASCO 2020 meeting. This included patients with R/R LBCL and follicular lymphoma (FL) after two or more lines of therapy. Prior exposure to CD19-directed therapy was allowed. The data were presented for 22 patients with age range 34–73 years, 64% with LBCL, and 41% with prior autologous stem cell transplant, and 18% with autologous CAR-T. The overall response rate (ORR) was 63%, with a 37% complete remission rate (CR) in the 19 evaluable patients. Retreatment was noted to be feasible. The most common grade ≥ 3 adverse events (AEs) were cytopenias. CRS occurred in 32% (7 patients), and none with grade ≥ 3; no dose-limiting toxicities or GvHD was observed [96•, 97]. This was further updated for 42 patients (infused 41; 11 LBCL, and 21 FL) at the ASCO 2021 meeting, including 9 patients that had previously received autologous CAR-T. The median time from enrollment to start of therapy was 5 days in 98% of the enrolled patients. The efficacy analysis was available for 32 patients, and ORR and CR rates were 75% (24) and 50% (16), respectively. Redosing led to clinical responses, with an overall treatment failure-free survival for autologous CAR-T naïve patients of 64% and 61% at 6 months for FL and LBCL. No dose-limiting toxicities or GvHD was observed, and one grade 3 neurotoxicity was reported. CRS occurred in 27% of patients, and none with grade ≥ 3. There were 5 treatment-emergent deaths, one each from pneumonia, arrhythmia, stroke, and two instances of COVID-19 infection. The ALPHA2 study (NCT04416984), an open-label, single-arm study of ALLO-501A in non-HLA, matched patients with R/R LBCL after two or more lines of treatment also presented at the ASCO 2021 meeting. Prior autologous CD19 CAR-T was allowed if the tumor remained CD19+. As of the April 19th, 2021 data cutoff, 13 patients were enrolled, and 12 patients were treated with ALLO-501A. One patient that developed central nervous system involvement before infusion was not treated. Nine patients evaluable for efficacy were CAR-T naïve, except for one who received prior autologous CAR-T and previously had a 16-week CR followed by relapse. Of the 9 patients eligible for efficacy analysis (*n* = 5, in consolidation cohort), both the ORR and CR rates were 56%. No dose-limiting toxicities, GvHD, or neurotoxicity was seen, and CRS occurred in 2 patients, both grade < 3 [98].

PBCAR0191, an off-the-shelf, allogeneic CAR-T product, is also being evaluated in the CD19+ R/R B cell NHL (NCT03666000). The preliminary safety and efficacy data from the ASCO 2021 meeting on 13 patients have been presented. The median time from eligibility confirmation to infusion was 6.5 days. No grade ≥ 3 CRS or neurotoxicity was observed, and no GvHD was noted either. The ORR at day+28 was 77% with a 54% CR rate [99]. The safety and efficacy of CTX110, an allo anti-CD19 CAR-T cell product modified by using CRISPR/Cas9 editing to disrupt the endogenous TRAC locus and disrupt β_2_M, which eliminates major histocompatibility complex (MHC) class I expression, are under evaluation in R/R LBCL, double/triple hit lymphoma, transformed FL, and FL grade 3b. The phase 1 CARBON trial (NCT04035434) is an open-label, multicenter, global study enrolling patients with 2 or more lines of treatment. Prior allogeneic stem cell transplant and autologous CD19 CAR-T are excluded. This trial is open and enrolling at this time, and the data are awaited [100].

## Implications of allogeneic CAR-T

Allogeneic “off the shelf” CAR-T offers the promise of a readily available, hopefully at a reduced cost and more accessible to the patients and resource-limited settings. In addition, the ability to generate CAR-Ts from healthy donor cells with better quality without exposure to prior chemotherapies or the immunosuppressive NHL microenvironment could be transformative. It would also allow for redosing and standardized scale production and quality maintenance. These could also permit targeting multiple antigens simultaneously or sequentially. Genome editing tools enable a broader application of these “off the shelf” products with multiplex gene knockout and targeted transductions focused on making them safer (less GvHD) and persistent (less allo rejection) for effective use. Still, many challenges remain in enhancing the efficacy of allo CAR-Ts, such as the degree of in vivo expansion and persistence of CAR-Ts for efficacy and effective lymphodepletion strategies, which is likely of greater importance in this setting. Also, the impact of allo CAR-T persistence on efficacy is yet to be determined as these are more likely to be eliminated by the recipient’s immune system. Redosing with the same or another allo CAR targeting different antigens may be the solution, carefully considering donor-specific antibodies. The association of persistence of CAR-T cells in aggressive lymphoma is still debatable, as autologous CAR-T with CD28 signaling domain (axi-cel) with shorter persistence can still ensue durable responses in aggressive lymphomas. On the other hand, redosing with the axi-cel in indolent lymphoma such as FL or marginal zone lymphoma (MZL) as done in the ZUMA-5 trial in 13 patients (11 FL, 2 MZL) showed an ORR rate of 100% and a 77% CR rate. The median duration of response after redosing had not been reached, and 8 and 4 patients developed CRS and neurotoxicity, respectively but no grade ≥ 3 [101]. Lastly, determination of the optimal subset of T cells, significantly enriched with less differentiated naïve, and stem cell-like memory cells would be critical for optimal expansion, survival, and long-term persistence. As more clinical data are generated by bringing these promising therapies to the bedside for efficacy and side effects, further advances will be made to create an ideal “off the shelf” CAR-T product.

## Conclusions

CAR-T has revolutionized the treatment landscape of lymphomas and other cancers. Developing low-cost, readily available, easily accessible, safe, and effective products using genome editing tools and non-alloreactive cells will help generate true “off the shelf products.” With the number of active investigations, more transformative updates are anticipated soon to advance the capacity of this novel therapy.
